# Immune remodeling after thymectomy in patients with anti-AChR antibodies

**DOI:** 10.3389/fimmu.2026.1920763

**Published:** 2026-09-23

**Authors:** Naoshi Koizumi, Makoto Kinoshita, Yoshiaki Yasumizu, Tomohiro Yata, Daisuke Motooka, Ryohei Yamamura, Itsuki Sano, Yu Suzuki, Akihiro Tanaka, Minami Kanakura, Toru Koda, Naoko Ose, Takashi Kanou, Kenji Kimura, Yasushi Shintani, Hideki Mochizuki, Tatsusada Okuno

**Affiliations:** 1 Department of Neurology, Graduate School of Medicine, The University of Osaka, Suita, Osaka, Japan; 2 Clinical Laboratory Department, National Hospital Organization Osaka Toneyama Medical Center, Toyonaka, Japan; 3 Department of Neurology, National Hospital Organization Osaka Toneyama Medical Center, Toyonaka, Japan; 4 Department of Neurology, Yale School of Medicine, New Haven, CT, United States; 5 Integrated Frontier Research for Medical Science Division, Institute for Open and Transdisciplinary Research Initiatives (OTRI), The University of Osaka, Suita, Osaka, Japan; 6 Genome Information Research Center, Research Institute for Microbial Diseases, The University of Osaka, Suita, Osaka, Japan; 7 Division of Health Sciences, Graduate School of Medicine, The University of Osaka, Suita, Osaka, Japan; 8 Department of General Thoracic Surgery, Graduate School of Medicine, The University of Osaka, Suita, Osaka, Japan

**Keywords:** myasthenia gravis, natural killer cells, single-cell RNA-sequence, thymectomy, thymoma

## Abstract

**Background:**

Thymectomy is an established treatment for thymoma-associated myasthenia gravis (MG). However, a subset of patients develop new-onset MG after surgery. Although thymectomy affects immune homeostasis, its comprehensive impact on the peripheral immune system remains unclear, particularly at the single-cell transcriptomic level. Therefore, we aimed to characterize immune remodeling following thymectomy in anti-acetylcholine receptor (AChR) antibody-positive patients without overt MG.

**Methods:**

Peripheral blood mononuclear cells were collected from three anti-AChR antibody-positive patients before and 7–22 months after thymectomy. Single-cell RNA and T cell receptor (TCR) sequencing were subsequently performed. Cell populations were annotated using SingleR and Azimuth. Differential gene expression was analyzed, followed by gene set enrichment analysis. TCR repertoire diversity was assessed using scRepertoire. Additionally, cell–cell communication was inferred using CellChat.

**Results:**

Thymectomy induced broad immune remodeling characterized by a relative reduction in naïve CD4^+^ and CD8^+^ T cells and a concomitant relative increase in natural killer (NK) cells, particularly the CD56^dim^ subset. Despite the relative decline in naïve T cells, activation-associated genes were upregulated and inflammatory pathways were enriched in the remaining cells, indicating an activation-associated transcriptional state. Monocyte subsets showed marked transcriptional changes, including upregulation of the expression of immediate early genes and downregulation of that of antigen presentation-related and inflammatory genes, consistent with substantial transcriptional remodeling. Furthermore, cell–cell communication analysis revealed a qualitative shift in the dominant signaling axis after thymectomy. Pre-thymectomy networks were characterized by TNF- and resistin-mediated signaling, whereas post-thymectomy networks were dominated by IFNγ signaling, primarily originating from CD56^dim^ NK cells and targeting monocyte subsets.

**Conclusions:**

Thymectomy induces coordinated remodeling of the peripheral immune system, which involves reduced thymic output, activation-associated transcriptional changes in T cells, relative increase in the proportion of NK cells, and transcriptional remodeling of monocytes. The dominant inflammatory signaling axis shifts from TNF/resistin-centered to IFNγ-centered communication after thymectomy. This bidirectional immune modulation may underlie both therapeutic effects and susceptibility to post-thymectomy MG, highlighting the importance of intercellular signaling dynamics in maintaining immune homeostasis.

## 1 Introduction

Myasthenia gravis (MG) is an autoimmune disease caused by antibodies against the acetylcholine receptor (AChR) at the neuromuscular junction (1). MG is frequently associated with thymoma; approximately 10–20% of patients with MG have thymomas. Moreover, MG is the most common autoimmune condition linked to thymomas, with a prevalence of 30–44% (2). Thymoma induces various autoimmune responses (3, 4). Although thymectomy is recognized as an effective treatment for thymoma-associated MG (TAMG) (5), cases of new-onset MG or exacerbation of MG following thymectomy have been reported (6, 7). Approximately 1–11% of patients with thymoma develop MG after thymectomy, with the interval between surgery and disease onset ranging from 4.5 to 52.1 months. The incidence of post-thymectomy MG is substantially higher among patients with thymoma who are seropositive for AChR antibodies before surgery, with 22.2–30.3% developing MG during follow-up. However, the duration of observation differs across studies (8). Furthermore, the comprehensive impact of thymectomy on the peripheral immune system remains unclear. Thymectomy reduces thymic output, as reflected by decreased T-cell receptor excision circles. Consequently, naïve T-cell populations decline in the peripheral blood, whereas memory T-cell populations may increase (9–11). In addition, thymus-derived B-cell clones persist in circulation after thymectomy. These clones exhibit features of antigen-driven maturation, including class switching and somatic hypermutation (12). In TAMG, populations of cytotoxic natural killer (NK) cells characterized as CD56^dim^ CD16^+^ were reduced. However, their count increased following thymectomy (13). In TAMG, peripheral blood plasmacytoid dendritic cell (pDC) counts—especially pDC1—were reduced at baseline and further decreased after thymectomy (14). Nevertheless, these observations were largely derived from analyses of selected cell populations or limited immunological markers. Comprehensive immune alterations following thymectomy have not been investigated at the transcriptomic level using single-cell RNA sequencing (scRNA-seq).

In this study, we aimed to characterize the immune response changes induced by thymectomy in anti-AChR antibody-positive patients without apparent MG symptoms. We further aimed to elucidate the pathophysiology of post-thymectomy MG.

## 2 Materials and methods

### 2.1 Patients

This study included three patients positive for anti-AChR antibody without apparent symptoms of MG. Details of the three patients are presented in **Table 1**. All patients were recruited from the University of Osaka hospital. The study protocol involving human participants was reviewed and approved by the Ethics Committee of Osaka University (approval number: 11298–40). The patients provided their written informed consent to participate in this study.

**Table 1:** Clinical characteristics of patients included in this study. .

Patient no.	Condition	Age at sample collection	Sex	WHO histological classification of thymoma	Masaoka–Koga stage	AChR antibody level (pmol/mL)	Time after thymectomy (month)	Comorbidities	Predonisolone
case1	pre thymectomy	59	M	B2	Stage III	5.3	NA	None	None
case1	post thymectomy	61	M	B2	Stage III	3.8	22	None	None
case2	pre thymectomy	58	F	B2	Stage II	15	NA	Breast cancer	None
case2	post thymectomy	59	F	B2	Stage II	15	9	Breast cancer	None
case3	pre thymectomy	41	F	B1	Stage II	2.8	NA	Ulcerative colitis	None
case3	post thymectomy	42	F	B1	Stage II	4.6	7	Ulcerative colitis	5mg/day

The table summarizes demographic and clinical information for the three patients who underwent thymectomy. Variables include age, sex, World Health Organization (WHO) histological classification of thymoma, Masaoka–Koga stage, anti-acetylcholine receptor (AChR) antibody levels, sampling time points relative to thymectomy, and treatment history. Pre- and post-thymectomy samples are indicated, and the time after surgery is shown in months. AChR, acetylcholine receptor; MG, myasthenia gravis.

### 2.2 Peripheral blood mononuclear cell isolation

Patient blood was collected twice: once before thymoma resection and at 22, 9, and 7 months after thymectomy in Cases 1, 2, and 3, respectively, as specified in **Table 1**. All pre- and post-thymectomy blood samples were collected and processed using the same standardized protocol. Blood was collected into sodium heparin tubes. Whole blood was diluted 1:1 with Hanks’ Balanced Salt Solution (HBSS) and gently mixed by pipetting approximately 10 times before being carefully layered onto Ficoll-Paque™ PLUS (Cytiva, Uppsala, Sweden). Samples were centrifuged at 700 × g for 20 min at room temperature, and the peripheral blood mononuclear cell (PBMC) layer at the interface was collected. The isolated PBMCs were sequentially washed with 30 mL and then 10 mL of HBSS and subsequently stored at −80 °C under identical storage conditions until analysis. For scRNA-seq, frozen PBMCs were gently thawed using RPMI medium supplemented with 10% fetal bovine serum (FBS), pre-warmed to 37 °C. The cells were gently resuspended by pipetting approximately five times over several minutes and then passed through a 40-μm cell strainer, followed by washing before further processing. Cell viability was assessed by Trypan blue exclusion and was ≥ 95% for all samples used for scRNA-seq. Paired pre- and post-thymectomy samples from each patient were thawed and processed simultaneously to minimize potential differences arising from sample handling.

### 2.3 scRNA-seq analysis

The preserved PBMC samples were gently thawed for scRNA-seq analysis. To minimize batch effects, paired samples collected before and after thymectomy were processed simultaneously. Single-cell gene expression and T cell receptor (TCR) libraries were prepared using the Chromium Next GEM Single Cell 5′ Reagent Kits v2 (10x Genomics) according to the manufacturer’s instructions. Libraries were sequenced on a NovaSeq X Plus platform (Illumina). An average of 28,000 read pairs per cell was obtained for each library. Sequencing data in FASTQ format were processed using Cell Ranger v8.0.1 or 9.0.1 (10x Genomics) with the GRCh38-2024-A reference. Gene expression libraries were analyzed to generate gene-barcode matrices, and V(D)J libraries were analyzed for TCR sequence assembly and clonotype assignment. The raw data have been deposited in the NCBI Gene Expression Omnibus database (GSE330576).

First, quality control (QC) and doublet removal were performed. Droplets with fewer than 500 or more than 20,000 unique molecular identifier (UMI) counts, or with fewer than 400 or more than 5,000 unique genes (features), were excluded to remove low-quality cells and potential doublets. Additionally, droplets in which more than 15% of UMI counts were mapped to mitochondrial genes were filtered out to eliminate stressed or dying cells. Potential doublets were identified and removed using the DoubletFinder package in R (version 2.0.6), together with Seurat (version 5.3.1). Homotypic doublet proportions were estimated based on Seurat cluster annotations, assuming a 5% expected doublet formation rate (15). Following QC and doublet removal, individual Seurat objects were normalized using the NormalizeData function, and highly variable features were identified using FindVariableFeatures with the “vst” method; 2,000 genes were selected. Processed datasets were then merged into a single Seurat object. The merged data were scaled with ScaleData and subjected to principal component analysis using the first 30 components. Harmony integration was performed with IntegrateLayers to correct for batch effects across samples, specifying “pca” as the original reduction and “harmony” as the new reduction. Clustering was conducted in the Harmony-reduced space using FindNeighbors and FindClusters with a resolution of 2, and visualization was performed using uniform manifold approximation and projection (UMAP) with RunUMAP based on the Harmony embedding. Initial cell-type annotation was performed using SingleR with the MonacoImmuneData PBMC reference (16, 17). To achieve finer immune subset classification and identify platelet populations not represented in the MonacoImmuneData reference, additional annotation was performed using Azimuth (18). Azimuth identified a distinct platelet cluster, the distribution of which is shown in **Supplementary Figure S1**. For analyses requiring refined immune cell classification, including NK cell subset characterization, monocyte subset analysis, and cell–cell communication analysis, Azimuth-based annotations were used.

### 2.4 Analysis of differentially expressed genes

Differentially expressed genes (DEG) between pre- and post-treatment conditions were analyzed for each annotated cell population using the Nebula package (v1.5.3) based on a negative binomial mixed model. Genes expressed in ≥10% of cells with total UMI counts ≥20 were tested, excluding ribosomal and mitochondrial genes. The model included patient ID and treatment condition as random and fixed effects, respectively; library size was incorporated as an offset. P-values were adjusted using the Benjamini–Hochberg method, and genes with a false discovery rate (FDR) < 0.05 and |log2FC| > 0.25 were considered significantly differentially expressed. QC filters were applied to retain genes with acceptable model convergence (convergence code > –20) and moderate overdispersion (cell-level overdispersion < 100) (19). Cell populations containing fewer than 50 cells in total were excluded from differential expression analysis because of insufficient cell numbers for reliable statistical analysis.

### 2.5 Gene set enrichment and pathway analyses

Gene set enrichment analysis (GSEA) was performed using the fgsea package in R. All genes were ranked based on the log2 fold change obtained from differential expression analysis, and enrichment was tested against Gene Ontology (GO) biological processes and Hallmark. Gene sets containing 15–500 genes were analyzed with 10,000 permutations. Pathways with FDR < 0.25 were considered significantly enriched (20–22).

### 2.6 Repertoire analysis

TCR clonality and diversity were analyzed using scRepertoire (v2.2.1) (23). Shannon and inverse Simpson diversity indices were calculated for overall and subset-specific T cell populations. Pre- versus post-treatment comparisons were conducted using paired t-tests (n = 3 patients) (24).

### 2.7 CD4^+^ T cell automatic labeling and quantification of gene programs

PBMC data were processed using a pipeline developed in a previous study (25, 26) to assign CD4^+^ T cell clusters. Briefly, Azimuth (18) was used for the initial annotation of PBMC subsets, and cells annotated as CD4^+^ T cell subsets were extracted for downstream analysis. The extracted CD4^+^ T cells were normalized using SCTransform and then mapped to a reference dataset using Symphony (25, 27), which assigned cells to five major clusters (L1: Tnaive, Tcm, Tem, Temra, and Treg) and 18 minor clusters (L2). To quantify functional gene program activity, non-negative matrix factorization projection (NMFproj) was applied to score each cell across 12 predefined gene programs. Changes in NMF scores between pre- and post-treatment conditions were assessed for each cluster using a generalized linear model with donor included as a covariate. P-values were corrected for multiple testing using the Benjamini–Hochberg method.

### 2.8 Cell-cell interaction

Cell–cell communication networks were inferred using the R package CellChat (28, 29) following the official workflow. Normalized count data from Seurat objects were converted into CellChat objects using the human ligand–receptor reference database CellChatDB.human. Overexpressed ligands and receptors were identified, and communication probabilities were computed according to the standard CellChat pipeline.

### 2.9 Statistical analysis

The relative frequency of each cell type was calculated for every sample and compared between pre- and post-treatment conditions. Cell-type proportions are expressed as percentages of total cells per sample and ordered by mean abundance across all samples. Visualization was performed using ggplot2 (version 3.5.2) in R (version 4.4.2).

## 3 Results

### 3.1 Immune clusters obtained from anti-AChR antibody positive patients

Three patients positive for anti-AChR antibody without apparent symptoms of MG were enrolled in this study. PBMCs were collected both preoperatively and postoperatively to observe changes resulting from thymoma resection in peripheral immune dynamics. Postoperative PBMCs were collected at 22, 9, 7 months after surgery (**Figure 1A**). scRNA-seq and TCR sequencing were performed after QC, and 93,714 cells were included. Furthermore, cell annotation was performed on the obtained cells (**Figure 1B**). The gene expression profiles of the cells are shown in **Figure 1C**.

**Figure 1:**
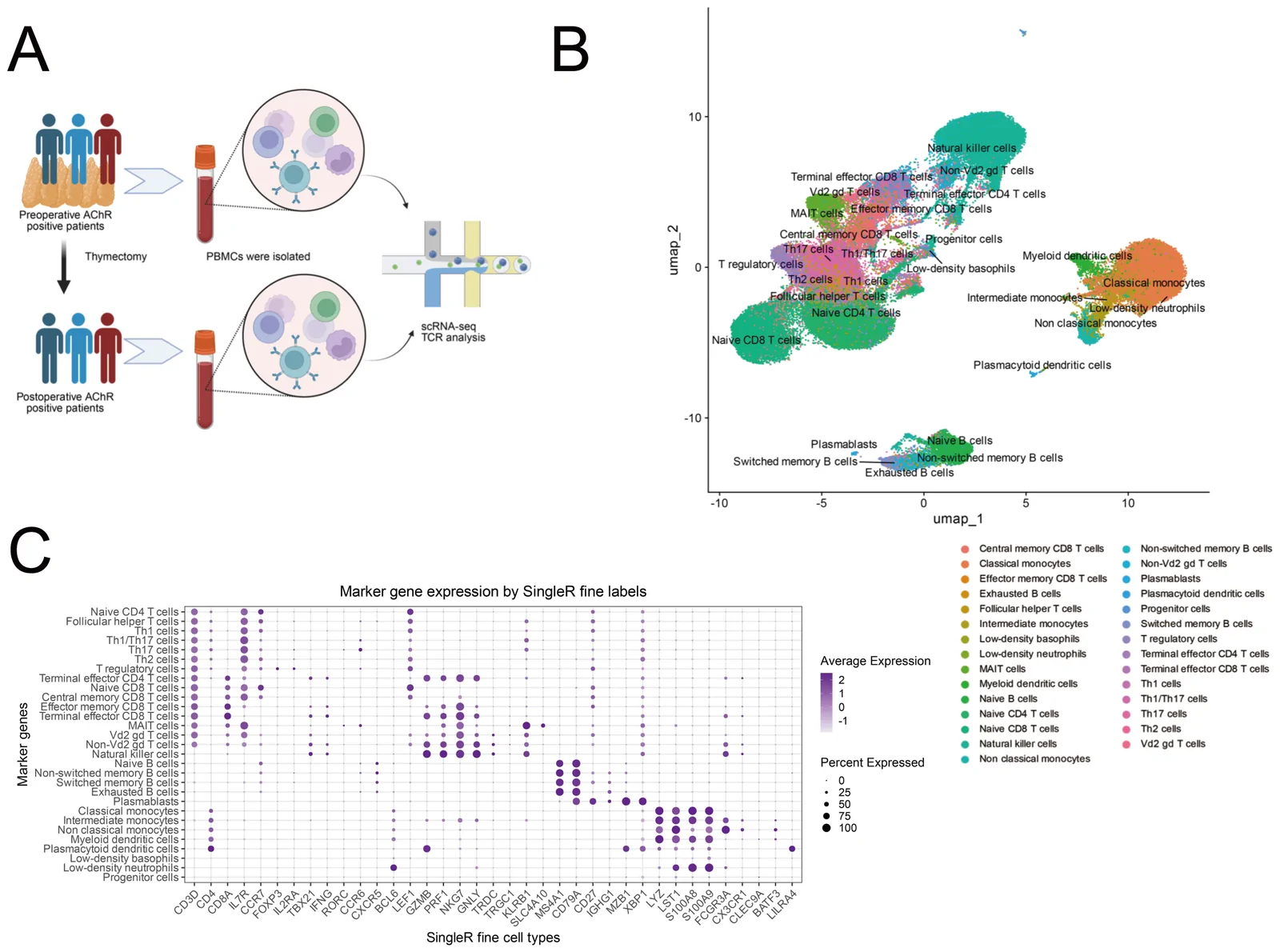
Study design and single-cell transcriptomic landscape of Peripheral blood mononuclear cells before and after thymectomy. **(A)** Schematic overview of the study design. Peripheral blood mononuclear cells (PBMCs) were collected from anti-acetylcholine receptor antibody-positive patients without apparent myasthenia gravis (MG) symptoms before and 7–22 months after thymectomy. PBMCs were isolated and subjected to single-cell RNA sequencing (scRNA-seq) and T cell receptor (TCR) repertoire analysis. Figures were created with biorender.com. **(B)** Uniform manifold approximation and projection (UMAP) visualization of 93,714 PBMCs integrated from all samples based on scRNA-seq data. Cells are colored according to fine cell-type annotations assigned using SingleR. **(C)** A dot plot showing expression of representative marker genes across SingleR-defined fine cell types.

### 3.2 Global changes in immune cell composition after thymectomy

When classified by major cell types, the PBMC populations included T cells (preoperative: 54–73%, postoperative: 40–67%), B cells (preoperative: 1.9–5.6%, postoperative: 1.1–10%), NK cells (preoperative: 7.4–18%, postoperative: 8.0–47%), monocytes (preoperative: 11–25%, postoperative: 9.1–29%), dendritic cells (preoperative: 0.68–1.4%, postoperative: 0.61–2.0%). In all three cases, the proportion of T cells tended to decrease, whereas that of NK cells tended to increase after thymectomy (**Figure 2A**). In the detailed classification by SingleR, the proportions of NK cells (preoperative: 7.4–18%, postoperative: 8.0–47%) and intermediate monocytes (preoperative: 0.58–2.5%, postoperative: 2.3–3.0%) tended to increase in all three cases. In contrast, the proportions of naïve CD4^+^ T cells (preoperative: 12–19%, postoperative: 7.5–18%), naïve CD8^+^ T cells (preoperative: 9.8–16%, postoperative: 5.0–13%), T helper type 2 (Th2) cells (preoperative: 2.4–8.3%, postoperative: 1.7–4.6%), regulatory T cells (Tregs) (preoperative: 2.3–4.6%, postoperative: 1.7–4.0%), terminal effector CD8^+^ T cells (preoperative: 1.8–7.2%, postoperative: 1.4–5.3%), mucosal-associated invariant T (MAIT) cells (preoperative: 0.29–5.8%, postoperative: 0.18–5.6%), and effector memory CD8^+^ T cells (preoperative: 0.37–0.47%, postoperative: 0.33–0.46%) tended to decrease after thymectomy in all three cases (**Figure 2B**). DEG analysis revealed substantial transcriptional changes after thymectomy across multiple immune cell types (**Figure 2C**). The number of DEGs varied markedly among cell types, with monocyte subsets showing the largest transcriptional alterations. In particular, intermediate monocytes and myeloid dendritic cells exhibited the highest numbers of DEGs, whose expression was predominantly upregulated after thymectomy.

**Figure 2:**
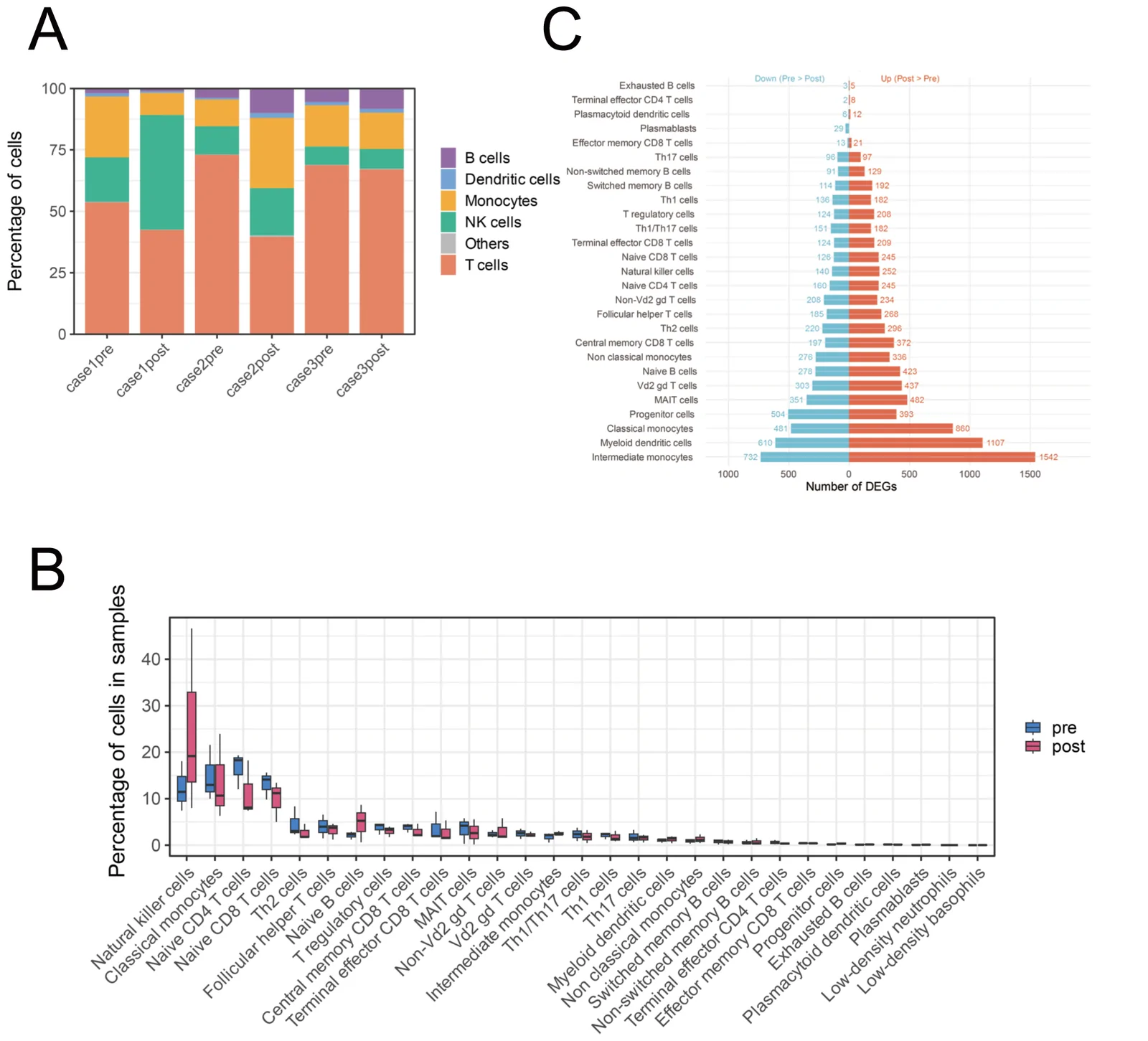
Changes in peripheral immune cell composition and transcriptional activity after thymectomy. **(A)** Stacked bar plots showing the relative proportions of major immune cell groups (T cells, B cells, natural killer cells, monocytes, dendritic cells, and others) defined by SingleR annotation in PBMCs from each patient before and after thymectomy. Cell-type proportions are expressed as percentages of total PBMCs per sample. **(B)** Box plots depicting the relative abundance of immune cell subsets defined by SingleR annotation in pre- and post-thymectomy samples. Each box represents the distribution of cell-type proportions across the three patients, with the median indicated by a horizontal line. Blue and pink denote pre- and post-thymectomy conditions, respectively. **(C)** A bar plot summarizing the number of differentially expressed genes (DEGs) identified in each immune cell type following thymectomy. Differential expression analysis was performed using a negative binomial mixed model, with genes considered significant at FDR < 0.05 and |log2 fold change| > 0.25. Blue and red bars indicate genes with downregulated (pre > post) and upregulated (post > pre) expression after thymectomy, respectively. The total number of DEGs across all analyzed cell types is shown at the bottom.

### 3.3 Remodeling of T cell characteristics after thymectomy

In this study, T cell populations consistently showed a decreasing trend after thymectomy. In particular, naïve CD4^+^ and CD8^+^ T cells exhibited relatively high proportions and a consistent decreasing trend. DEG analysis revealed significant transcriptional changes in both naïve CD4^+^ and CD8^+^ T cells after thymectomy (**Figures 3A, B**). The genes with upregulated expression in both subsets included immediate early and activation-related genes such as *CD69, DUSP1*, *FOS*, *JUNB*, and *TNFAIP3*. Contrastingly, the expression of several regulators involved in transcriptional repression and cellular stress responses, including that of *HEXIM1*, *H2AX*, and *GADD45B*, was downregulated. Th2 cells, Tregs, and MAIT cells also showed a decreasing trend in all three cases after thymectomy. Supplementary differential expression analyses further supported the robustness of the observed transcriptional changes (**Supplementary Figures S2A–C**). Across all analyses, the expression of activation-associated genes was consistently upregulated in the postoperative samples, including that of immediate early genes such as *CD69*, *DUSP1*, and *FOS*. Contrastingly, the expression of genes involved in transcriptional repression and cellular stress responses was downregulated.

**Figure 3:**
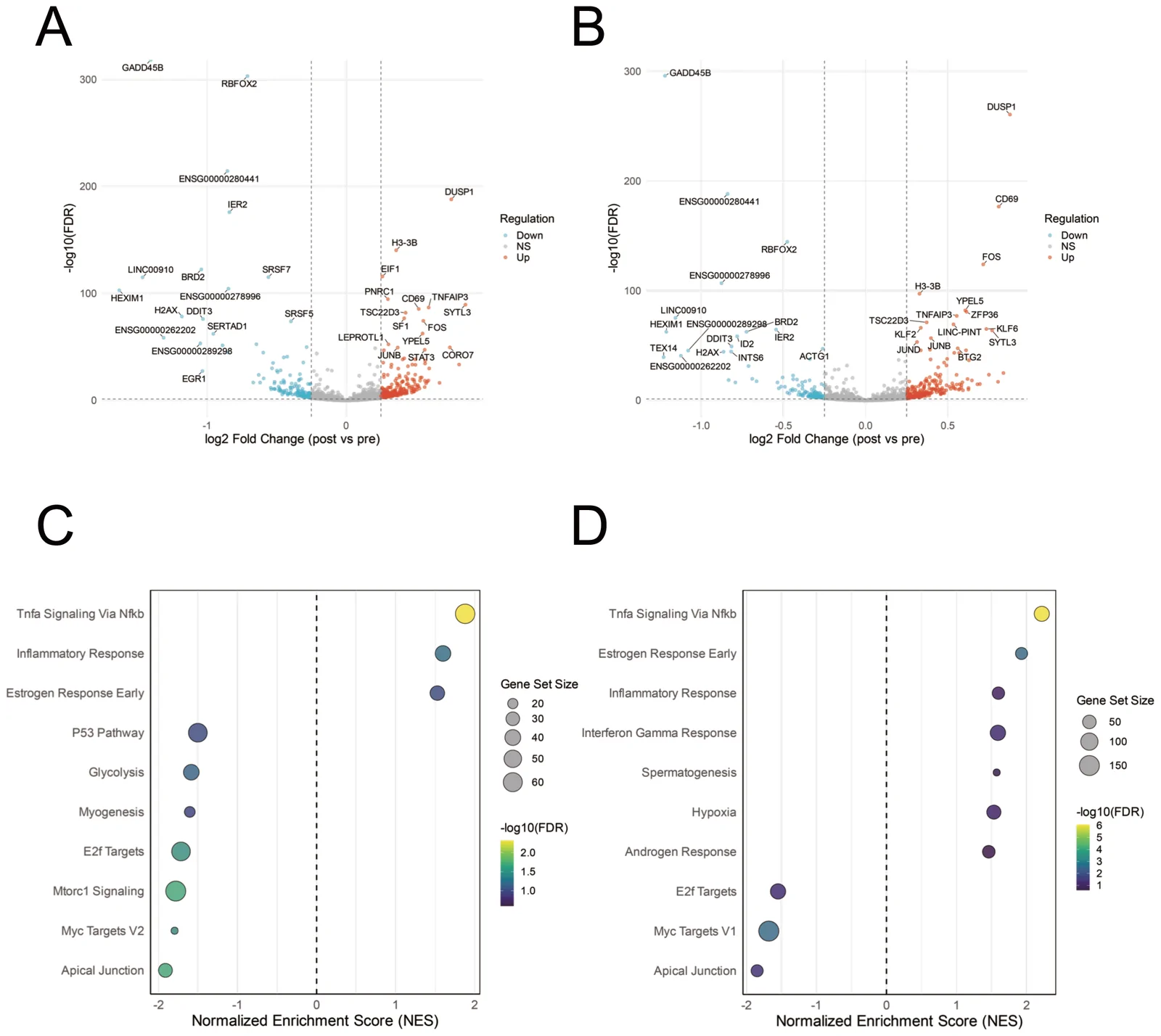
Transcriptional and pathway-level changes in naïve CD4^+^ and CD8^+^ T cells after thymectomy. **(A)** A volcano plot showing DEGs in naïve CD4^+^ T cells annotated by SingleR between post- and pre-thymectomy samples, as identified using a negative binomial mixed model. Genes with significantly upregulated or downregulated expression after thymectomy (FDR < 0.05, |log2FC| > 0.25) are highlighted in red and blue, respectively. Gray dots indicate non-significant genes. Selected genes are labeled. **(B)** A volcano plot of DEGs in naïve CD8^+^ T cells annotated by SingleR, displayed using the same criteria as in **(A)**. **(C)** Comparative gene set enrichment analysis (GSEA) of Hallmark pathways in naïve CD4^+^ T cells under post- and pre-thymectomy conditions. Pathways with FDR < 0.25 are shown. Positive values in the x-axis indicate enrichment after thymectomy. Dot size reflects gene set size, and color intensity corresponds to −log10(FDR). **(D)** Hallmark pathway GSEA for naïve CD8^+^ T cells, displayed as in **(C)**.

GSEA of Hallmark pathways revealed distinct transcriptional changes in naïve CD4^+^ and CD8^+^ T cells after thymectomy (**Figures 3C, D**). In naïve CD4^+^ T cells, inflammatory pathways, including TNFα signaling via NF-κB and inflammatory response, were positively enriched after surgery. In contrast, pathways related to cell cycle progression and cellular metabolism, such as E2F targets, mTORC1 signaling, and glycolysis, were negatively enriched. Similarly, naïve CD8^+^ T cells showed positive enrichment of TNFα signaling via NF-κB, interferon gamma response, and inflammatory response after thymectomy. In contrast, pathways related to cell proliferation and growth, including E2F targets and MYC targets, were negatively enriched.

Clonal size distribution and diversity indices were compared between preoperative and postoperative PBMC samples to assess the impact of thymectomy on TCR repertoire diversity. Analysis of clonal size distribution revealed no marked shifts in overall clonal composition after thymectomy across the three cases (**Supplementary Figure S3A**). Moreover, Shannon diversity analysis demonstrated no statistically significant differences in overall TCR repertoire diversity between preoperative and postoperative PBMC samples (**Supplementary Figure S3B**). When stratified by T cell subsets, including naïve CD4^+^ T cells, naïve CD8^+^ T cells, MAIT cells, Tregs, and helper T cells, no significant changes in Shannon diversity were detected after thymectomy (**Supplementary Figure S3C**). Consistent with the Shannon diversity results, inverse Simpson diversity indices showed no significant differences between preoperative and postoperative samples at the PBMC level (**Supplementary Figure S4A**). Similarly, subset-specific analyses revealed no statistically significant alterations in inverse Simpson diversity across multiple T cell populations following thymectomy (**Supplementary Figure S4B**). Together, these results indicate that thymectomy did not induce overt changes in TCR clonal diversity or repertoire evenness in peripheral blood T cells within the observed time frame.

We further examined CD4^+^ T cells more comprehensively by focusing on minor cluster-level (L2) subpopulations defined using Symphony reference mapping. At the L2 level, the proportions of TcmTfh, TemraTh1, TemTh1, and TemTph subsets among CD4^+^ T cells tended to increase after thymectomy across all three cases, whereas those of TnaiveMX1 and TnaiveSOX4 subsets consistently decreased (**Figure 4A**). Furthermore, the overall proportion of total Tregs decreased after thymectomy (**Figure 2B**). When Tregs were further subdivided into TregAct, TregEff, and TregNaive subsets, no consistent trend was observed (**Figure 4A**). We subsequently assessed changes in the gene program activity quantified by NMF projection across L2 subpopulations. Reduced activity was observed for NMF8 (Cent.Mem.-F) and NMF9 (Thy.Emi.-F), whereas NMF10 (Tissue-F) showed increased activity following thymectomy (**Figure 4B**). In addition, a marked increase was selectively observed in effector Tregs (TregEff) within the NMF5 (TregEff/Th2-F) program.

**Figure 4:**
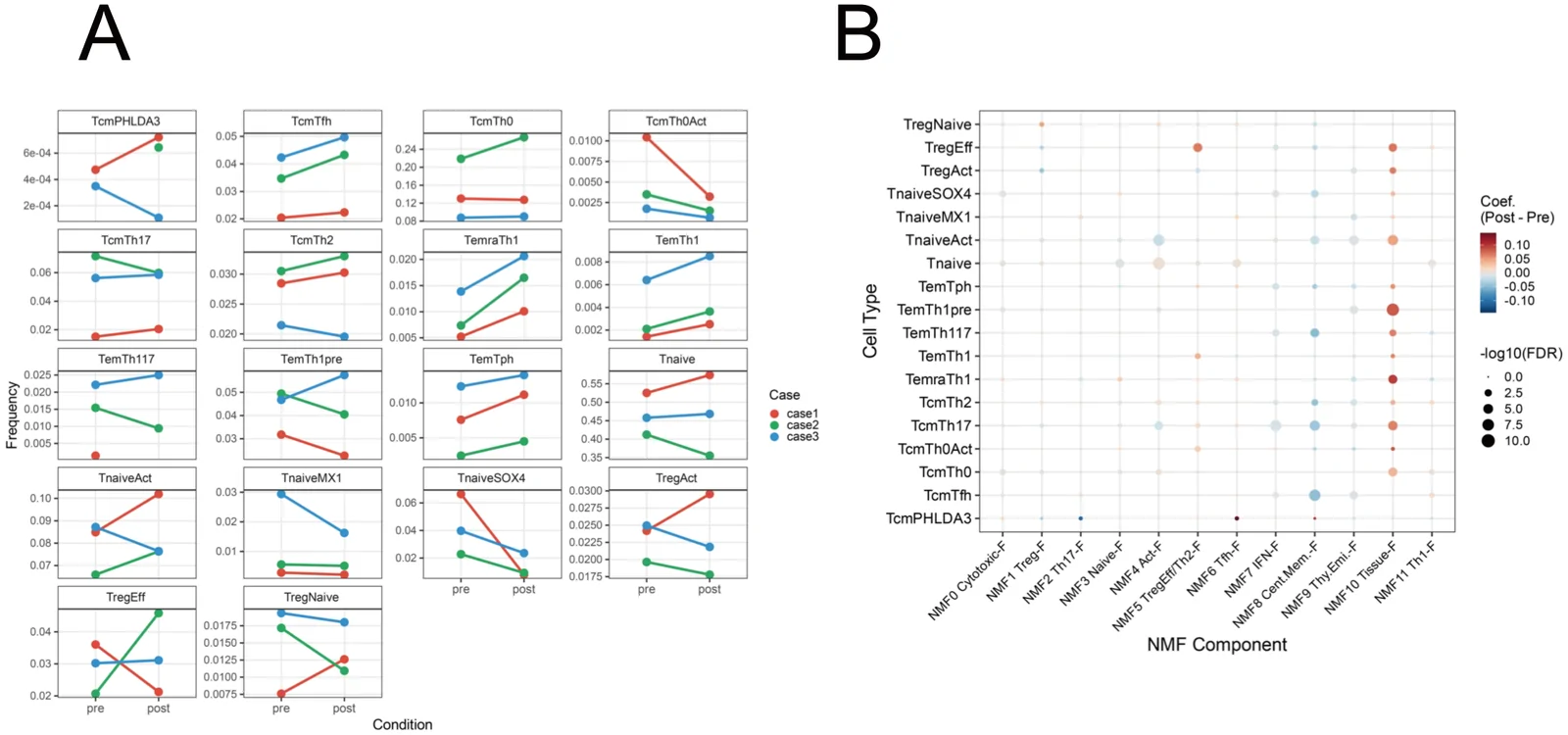
CD4^+^ T cell subset dynamics and gene program changes after thymectomy. **(A)** Frequencies of CD4^+^ T cell L2 subpopulations defined by Symphony reference mapping, shown for each case before and after thymectomy. Each panel represents one CD4^+^ T cell subset, and lines connect paired pre- and post-thymectomy samples from the same patient (cases 1–3). **(B)** Alterations of gene program activity quantified by non-negative matrix factorization projection (NMFproj) across CD4^+^ T cell L2 subpopulations after thymectomy. Dot plots depict changes in NMF-defined cell features for each subpopulation. Dot colors indicate regression coefficients (post minus pre), and dot sizes represent the statistical significance of generalized linear model (GLM) testing (−log10 FDR). Gene programs were annotated according to the original NMF definitions as follows: NMF0 [cytotoxic-feature (cytotoxic-F); GZMB, CX3CR1], NMF1 [Treg-feature (Treg-F); FOXP3, IL2RA], NMF2 [Th17-feature (Th17-F); RORC, CCR6], NMF3 [naive-feature (naive-F); CCR7, BACH2], NMF4 [activation-feature (Act-F); DACT1, CDK6], NMF5 [Treg effector/Th2-feature (TregEff/Th2-F); HLA-DR genes, CCR10], NMF6 [Tfh-feature (Tfh-F); MAF, CXCR5], NMF7 [interferon-feature (IFN-F); OAS1, MX1], NMF8 [central memory-feature (Cent.Mem.-F); CRIP2, PLP2], NMF9 [thymic emigrant-feature (Thy.Emi.-F); SOX4, PECAM1], NMF10 [tissue-feature (Tissue-F); JUNB, NFKBIA], and NMF11 [Th1-feature (Th1-F); GZMK, EOMES].

### 3.4 Relative increase and transcriptional remodeling of CD56^dim^ NK cells after thymectomy

We further analyzed the innate immune system. The NK cell population was re-annotated using Azimuth to determine which subset was increased after thymectomy (**Figure 5A**; **Supplementary Figure S5A**). UMAP visualization confirmed clear segregation of NK cell subsets, including CD56^dim^, CD56^bright^, and proliferating NK cells (**Figure 5A**). Quantitative analysis revealed an increase in the proportion of CD56^dim^ NK cells after thymectomy in all three cases, whereas changes in the CD56^bright^ subset were variable (**Figure 5B**). Given the relative increase of the NK CD56^dim^ population, we subsequently focused on this subset to characterize transcriptional changes associated with thymectomy. The expression of canonical activation- and immediate early-associated genes was upregulated, including that of *FOS*, *DUSP1*, *TNFAIP3*, and *CD69*. In addition, the expression of *ZNF331* and *SYTL3* was strongly upregulated, indicating pronounced transcriptional remodeling in CD56^dim^ NK cells after thymectomy (**Figure 5C**). GSEA was performed to further investigate pathway-level alterations. Hallmark pathway analysis revealed positive enrichment of inflammatory and stress-related pathways, including TNFα signaling via NF-κB, hypoxia, and early estrogen response, in CD56^dim^ NK cells after thymectomy (**Supplementary Figure S5B**). Consistent with these findings, GSEA based on Gene Ontology biological processes demonstrated enrichment of immune signaling-related pathways, including endolysosomal Toll-like receptor signaling and Fc receptor-mediated stimulatory signaling, as well as processes related to energy homeostasis and response to hydrogen peroxide in postoperative CD56^dim^ NK cells (**Figure 5D**). Together, these results indicate that thymectomy is associated with a relative increase of the NK CD56^dim^ population accompanied by coordinated transcriptional remodeling that is characterized by enhanced activation- and inflammation-associated gene expression programs.

**Figure 5:**
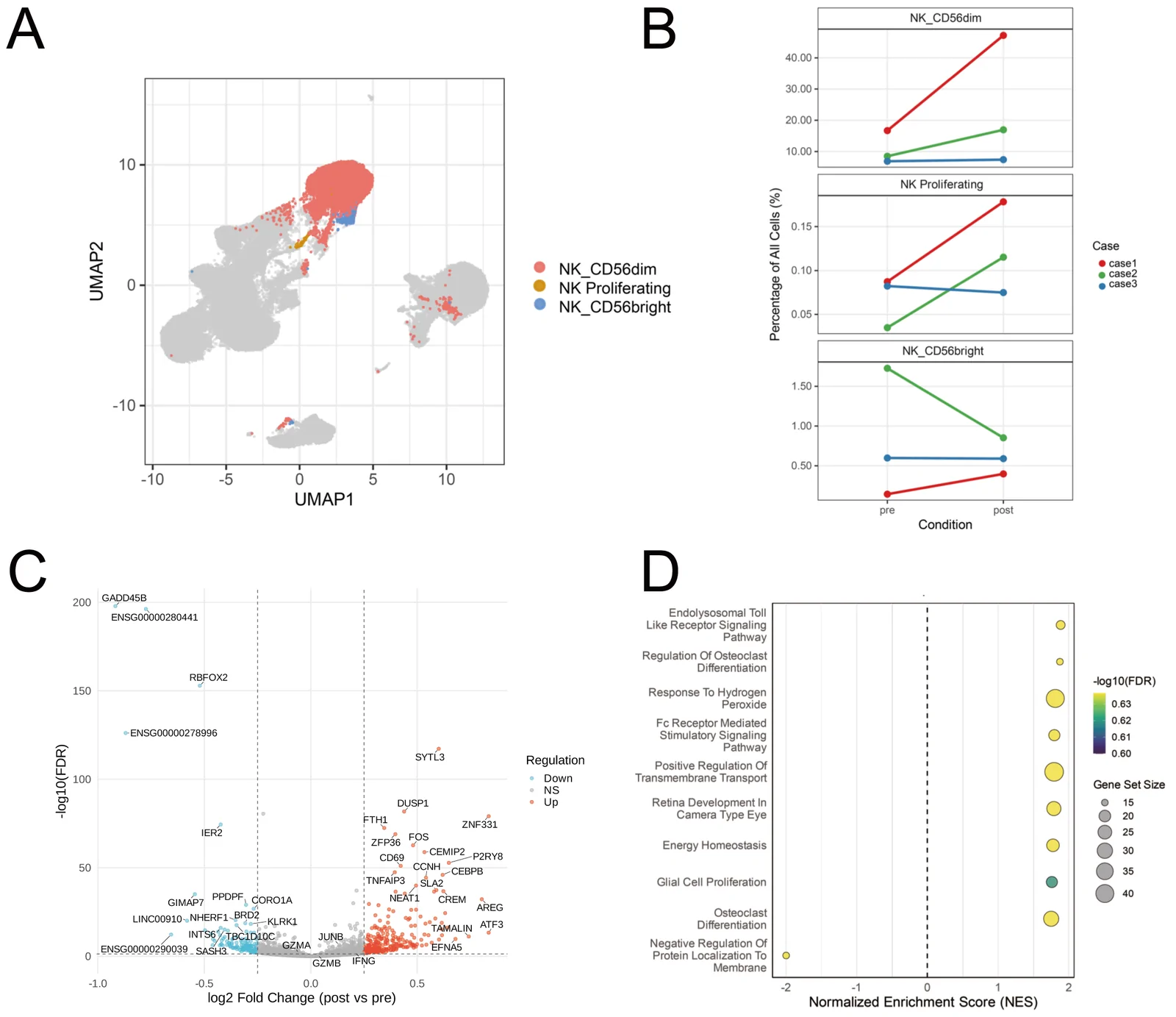
Azimuth-based annotation and transcriptional profiling of NK cell subsets before and after thymectomy. **(A)** UMAP highlighting NK cell subsets annotated using Azimuth. The Azimuth-defined NK population, which corresponds to conventional CD56^dim^ NK cells based on marker gene expression, is denoted as NK_CD56dim. Proliferating and CD56^bright^ NK cell subsets are shown as annotated by Azimuth. **(B)** Changes in the relative proportions of NK cell subsets (NK_CD56dim, NK proliferating, and NK_CD56bright) in pre- and post-thymectomy samples for each patient. **(C)** A volcano plot showing DEGs in CD56^dim^ NK cells between post- and pre-thymectomy samples, as identified using a negative binomial mixed model. Genes with significantly upregulated or downregulated expression after thymectomy (FDR < 0.05, |log2FC| > 0.25) are highlighted in red and blue, respectively. Gray dots indicate non-significant genes. Selected genes are labeled. **(D)** Gene Ontology biological process enrichment analysis of DEGs in NK_CD56dim cells. Dot size represents gene set size, and color indicates –log10 (FDR). Normalized enrichment scores (NES) are shown for pathways enriched in post- versus pre-thymectomy samples.

### 3.5 Transcriptional remodeling of monocyte subsets after thymectomy

Because the Monaco Immune reference used for initial SingleR annotation does not include platelet or megakaryocyte populations (**Supplementary Figure S1**), we next examined transcriptional changes in monocyte subsets using Azimuth-based annotations, which provide a platelet reference and have been demonstrated to robustly distinguish classical (CD14^+^ Mono) and non-classical (CD16^+^ Mono) monocyte populations. The expression of immediate early and activation-associated genes, including *FOS*, *FOSB*, *JUND*, *DUSP1*, and *ZFP36*, was consistently upregulated following thymectomy. In contrast, the expression of genes involved in interferon signaling and antigen presentation, such as *IFIT2*, *CD74*, *HLA-DRB5*, and *TNF*, was downregulated after surgery (**Figure 6A**). Non-classical monocytes also exhibited substantial transcriptional remodeling after thymectomy. The expression of *G0S2*, *CCL5*, and *NR4A2* was significantly upregulated, whereas that of *TNF*, *CD74*, *MNDA*, and *GADD45B* was downregulated. In addition, stress- and activation-responsive transcriptional regulators such as *ZFP36*, *JUNB*, *FOS*, and *FOSB* were prominently induced, indicating enhanced immediate-early gene activity in this subset (**Figure 6B**).

**Figure 6:**
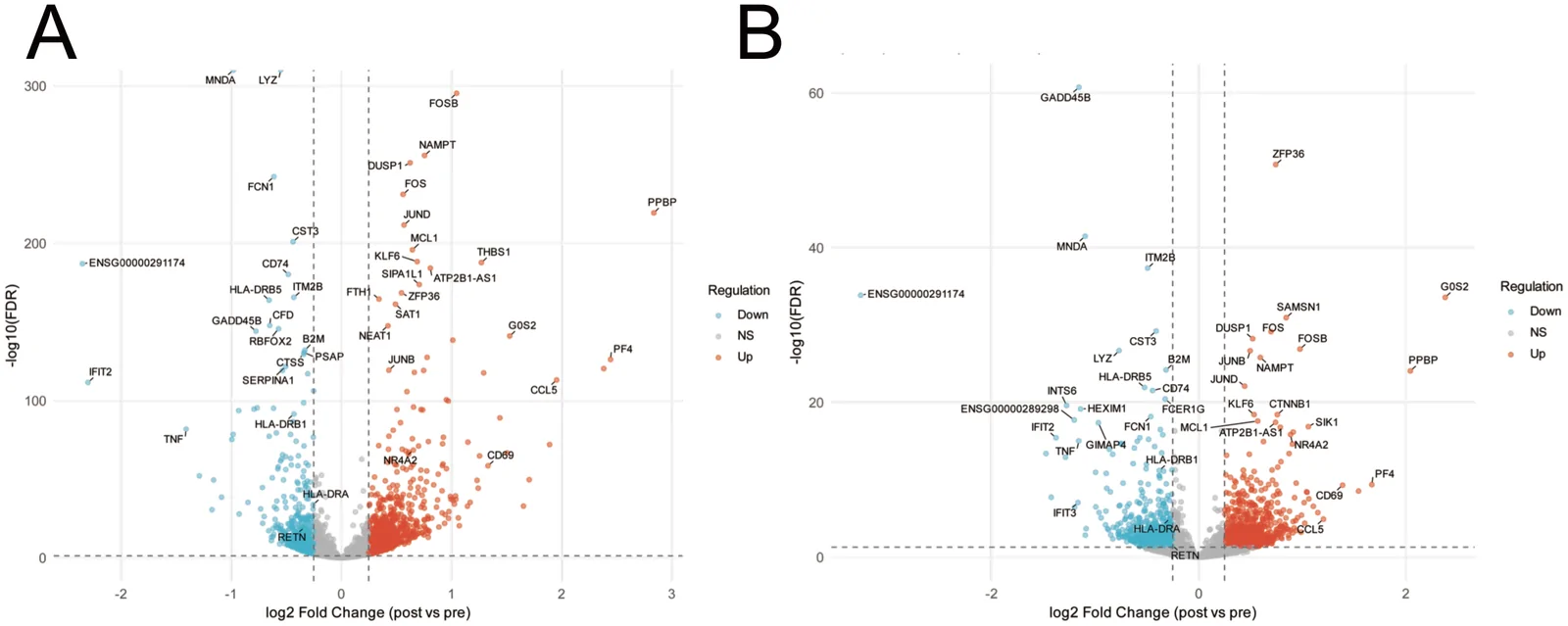
Transcriptional changes in monocytes after thymectomy. **(A)** A volcano plot showing DEGs in classical monocytes annotated by Azimuth, comparing post- to pre-thymectomy samples. Differential expression analysis was performed using a negative binomial mixed model. Genes with FDR < 0.05 and |log2 fold change| > 0.25 were considered significant. Red and blue dots indicate genes with upregulated and downregulated expression, respectively, after thymectomy; gray dots indicate non-significant genes. Selected genes are labeled. **(B)** A volcano plot of DEGs in non-classical monocytes annotated by Azimuth, displayed using the same criteria as in **(A)**.

### 3.6 Remodeling of the immune network after thymectomy

We performed ligand–receptor interaction analysis using Azimuth-based cell type annotations to characterize global changes in intercellular communication following thymectomy, which enabled refined classification of NK cell subsets. Analysis of overall signaling activity revealed a marked redistribution of sender and receiver roles after thymectomy (**Supplementary Figures S6A, B**). Monocyte subsets showed a reduced contribution as both signal senders and receivers in the postoperative condition. In contrast, plasmablasts, Tregs, and CD56^dim^ NK cells primarily exhibited increased signaling activity as signal receivers after thymectomy (**Supplementary Figures S6A, B**). Examination of individual ligand–receptor pairs further highlighted qualitative differences between pre- and post-thymectomy conditions (**Figures 7A, B**). TNF-related signaling pathways were dominant in preoperative samples, with strong interactions involving TNFSF4–TNFRSF4 and TNF–TNFRSF1A, as well as resistin-mediated signaling characterized by RETN–CAP1 and RETN–TLR4 interactions. In contrast, postoperative samples showed a relative increase in IFNγ-mediated signaling, characterized by enhanced IFNG–IFNGR1–IFNGR2 interactions. Consistent with these findings, both TNF and resistin signaling were globally increased in the pre-thymectomy condition, whereas type II interferon signaling was preferentially enriched after thymectomy (**Figures 7A, B**).

**Figure 7:**
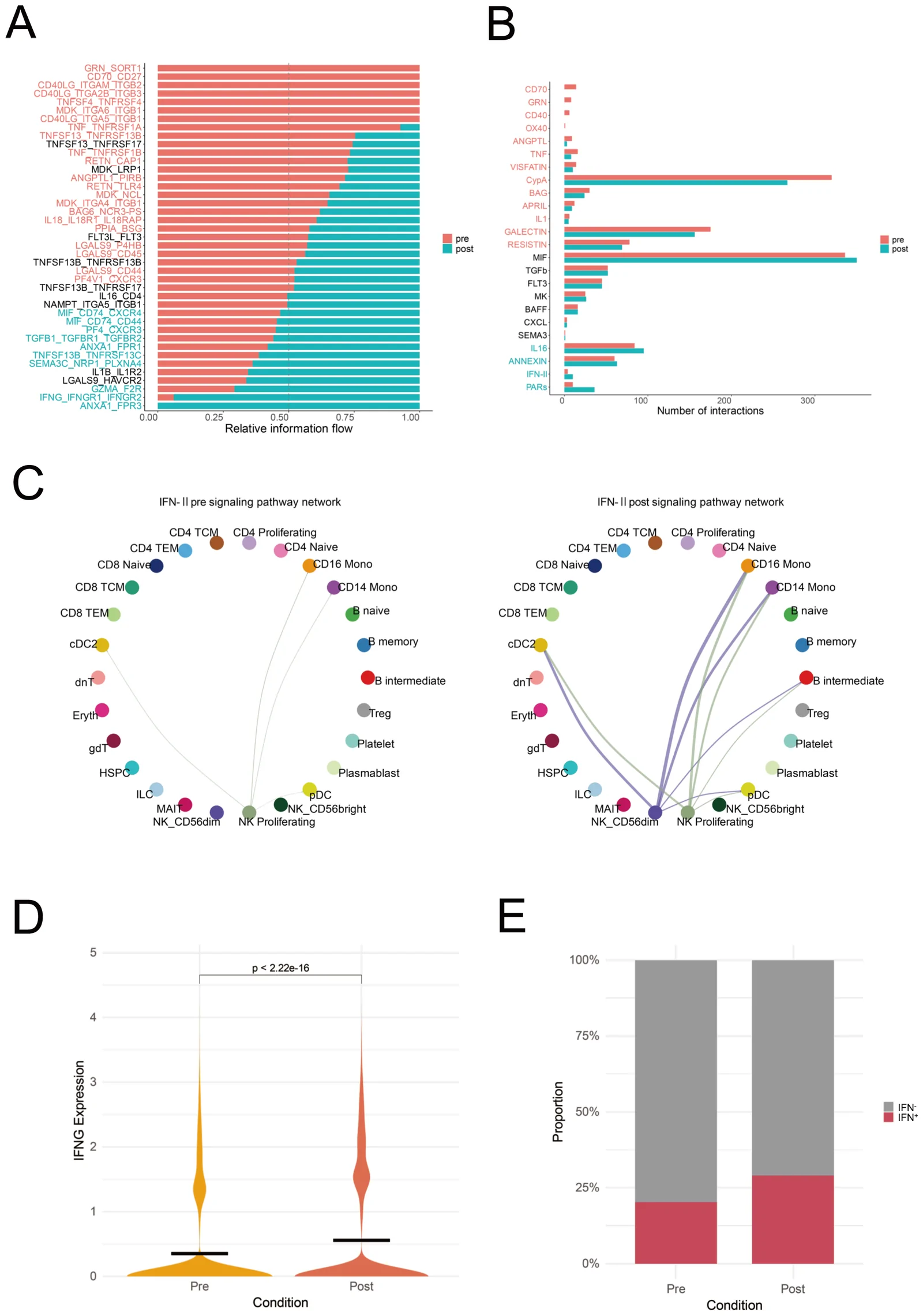
Reorganization of IFNγ signaling and cell–cell communication following thymectomy. **(A)** Bar plots ranking signaling pathways by relative information flow (interaction strength/weight) in pre- and post-thymectomy conditions, displayed in a stacked format. Pathways are ordered by overall contribution to communication, and statistical analysis was performed as implemented in CellChat. **(B)** Bar plots showing the number of interactions for each signaling pathway in pre- and post-thymectomy conditions in a non-stacked format, summarizing pathway-level differences in signaling activity. **(C)** Cell–cell communication networks of the IFN-II signaling pathway, as inferred by CellChat in pre- (left) and post-thymectomy (right) samples. Nodes represent immune cell types, and directed edges indicate ligand–receptor interactions. Edge thickness reflects the relative strength of inferred cell–cell communication based on ligand–receptor expression. **(D)** Violin plots showing single-cell IFNG expression levels in NK_CD56dim cells before and after thymectomy. Normalized IFNG expression values were obtained from the Seurat object (log-normalized expression from the RNA assay). Violin shapes depict the distribution of expression values across individual cells, and black crossbars indicate the mean expression level for each condition. Statistical significance was assessed using a Wilcoxon rank-sum test, and the resulting p-value is shown. **(E)** Bar plots showing the proportion of IFNG-expressing (IFNG^+^) and non-expressing (IFNG^-^) cells within the NK_CD56dim population before and after thymectomy. Cells were classified as IFNG^+^_if IFNG expression was detectable (log-normalized expression > 0) in the single-cell RNA-seq data; cells with no detectable expression were classified as IFNG^-^. Proportions were calculated within each condition (Pre and Post), and bars represent stacked fractions of IFNG^+^ and IFNG^-^ cells.

Based on the observed global remodeling of cell–cell communication after thymectomy, we subsequently evaluated signaling pathways that exhibited differential activity between pre- and post-thymectomy conditions. Analysis of sender–receiver relationships revealed that CD56^dim^ NK cells constituted the primary source of IFNγ signaling in postoperative samples, with increased signal transmission toward multiple monocyte subsets. In contrast, this signaling axis was weaker in pre-thymectomy samples (**Figure 7C**). Analysis of pathway-level signaling activity, focusing on pathways for which CD56^dim^ NK cells acted as the primary sender population, revealed distinct patterns between the two conditions. In pre-thymectomy samples, signaling pathways mediated by IL16, GZMA, and MIF were relatively enriched. In contrast, postoperative samples showed increased activity of pathways mediated by IFNG and TGFB1, indicating a qualitative shift in dominant cytokine-mediated communication after thymectomy (**Supplementary Figure S6C**). At the gene expression level, IFNG expression was increased after thymectomy in CD56^dim^ NK cells, accompanied by an increase in the number of IFNG-expressing cells (**Figures 7D, E**).

Additional pathway-specific analyses showed that resistin signaling, which was mainly derived from monocyte subsets in the pre-thymectomy condition, was reduced after surgery. Similarly, TNF-mediated signaling, mainly originating from CD16^+^ monocytes and other monocyte subsets before thymectomy, was attenuated in the postoperative samples. In contrast, TGFβ signaling showed no substantial change in either signal strength or cellular sources following thymectomy (**Supplementary Figure S7**).

## 4 Discussion

In this study, we performed a comprehensive single-cell transcriptomic analysis of peripheral immune cells before and after thymectomy in anti-AChR antibody–positive patients without overt MG. By integrating cell composition analysis, differential gene expression, pathway enrichment, TCR repertoire profiling, and cell–cell communication inference, we provide an unbiased overview of thymectomy-induced immune remodeling.

T cell subsets were annotated using reference-based mapping (SingleR based on the MonacoImmuneData PBMC reference) (16) and further supported by the expression of canonical markers such as CCR7. A recent consensus guideline has emphasized that T cell subset definitions should be explicitly described based on the experimental criteria used in each study, particularly in the context of high-dimensional single-cell analyses (30). Our classification follows these principles. Therefore, it should be interpreted with the understanding that T cell phenotypes likely exist along a continuum rather than as strictly discrete subsets. Consistent with previous reports, we observed a relative reduction in naïve CD4^+^ and CD8^+^ T cell populations after thymectomy (10). Given that thymomas and the thymus are known sources of newly generated T cells (11, 31), particularly recent thymic emigrants, this finding likely reflects reduced thymic output following surgical resection. This interpretation is further supported by the consistent relative decrease in the TnaiveSOX4 subpopulation, which is enriched for recent thymic emigrant-like cells (32). Despite the reduction in naïve T cell counts, DEG and pathway analyses revealed a coordinated upregulation of the expression of immediate early and activation-associated genes, including *FOS*, *JUNB*, *DUSP1*, and *CD69* (33–35), alongside enrichment of inflammatory signaling pathways such as TNFα signaling via NF-κB and interferon gamma response. Importantly, *CD69* expression is not exclusively indicative of antigen-driven TCR activation but can also reflect cytokine-mediated or homeostatic activation (36). Together, these findings indicate an activation-associated transcriptional state in the remaining naïve T cells after thymectomy. This pattern may reflect a homeostatic transcriptional response to reduced thymic output, although its functional significance remains to be determined (37). Kohler et al. (38) suggested that thymectomy contributes to the development of autoimmune diseases by increasing the proportion of Tregs with reduced suppressive capacity. However, detailed subclassification of Tregs did not reveal a consistent trend in our analysis, making it difficult to draw definitive conclusions. Importantly, TCR repertoire diversity remained largely preserved, indicating that thymectomy did not induce overt clonal restriction within the observation period. This dissociation between numerical decline and repertoire diversity demonstrates that peripheral homeostatic mechanisms may mitigate the impact of reduced thymic output.

In parallel with the relative reduction in T cell populations, we observed a consistent increase in the relative proportion of NK cells after thymectomy, particularly within the CD56^dim^ subset. Although NK cell development primarily occurs in the bone marrow and is largely independent of the thymus (39), this finding is consistent with previous observations in TAMG (13) and suggests that thymectomy alters the balance between adaptive and innate immunity. In the present study, the expression of activation-related and immediate early genes, including *FOS*, *DUSP1*, and *CD69*, was upregulated in CD56^dim^ NK cells (35, 40). The induction of these genes indicates substantial transcriptional remodeling following thymectomy. Pathway analyses further demonstrated enrichment of inflammatory and stress-response pathways, supporting substantial transcriptional remodeling of CD56^dim^ NK cells after thymectomy. These findings may reflect an altered balance between innate and adaptive immune compartments following thymectomy.

Monocyte subsets exhibited broad transcriptional changes after thymectomy. The expression of immediate early genes (*FOS*, *FOSB*, *JUNB*, and *DUSP1*) was robustly upregulated across classical and non-classical monocytes, consistent with activation-associated transcriptional changes (41). Simultaneously, the expression of genes associated with antigen presentation, interferon signaling, and inflammatory response, such as *CD74*, HLA class II genes, *IFIT2*, and *TNF*, was consistently downregulated. This result is not contradictory to previous reports, given that TNF has been shown to enhance antigen-presenting capacity (42). These findings suggest that monocytes adopt a transcriptional state characterized by reduced expression of genes associated with inflammatory responses and antigen presentation after thymectomy. Monocytes in patients with manifest MG exhibit enhanced inflammatory responses and increased antigen-presenting activity (43). Thus, the reduced expression of inflammatory and antigen-presentation-related genes in monocytes may represent one component of the attenuation of monocyte-associated inflammatory programs after thymectomy. The increased expression of platelet-associated transcripts such as PF4 and PPBP should be interpreted cautiously. These signals may reflect platelet-derived transcripts, platelet-containing multiplets, or ambient platelet RNA rather than monocyte-intrinsic transcriptional activation.

Cell–cell communication analysis further indicated a marked shift in the dominant intercellular signaling landscape after thymectomy. Before thymectomy, signaling networks were largely centered on monocyte-derived inflammatory pathways, particularly TNF- and resistin-related interactions. In contrast, the communication network became dominated by IFNγ signaling post-thymectomy. CD56^dim^ NK cells emerged as the principal sender population, whereas multiple monocyte subsets acted as major receivers. This switch was supported by both the pathway-level analysis and the increased IFNG expression and proportion of IFNG-expressing cells in CD56^dim^ NK cells after thymectomy. IFNγ has been implicated in the initiation and exacerbation of MG in previous studies (44–48). In addition, its expression is elevated in the thymic microenvironment of TAMG (49). Excessive IFNγ signaling can promote antigen presentation, inflammatory cytokine production, and tissue damage (50). In contrast, both TNF and resistin are elevated in MG and participate in inflammatory disease processes (51, 52). Therefore, our findings suggest that thymectomy exerts bidirectional immunological effects: attenuation of TNF- and resistin-mediated signaling may represent a therapeutically beneficial aspect of immune remodeling, whereas enhancement of NK cell-derived IFNγ signaling may contribute to immune dysregulation and susceptibility to post-thymectomy MG. Consistent with this interpretation, TGFβ signaling remained largely unaltered after thymectomy. Together, these findings suggest that thymectomy induces a qualitative reorganization of intercellular communication networks rather than a uniform increase or decrease in inflammatory activity, highlighting a shift in the dominant signaling axis.

Previous studies have highlighted interactions between inflammatory signaling and thymic function, with inflammation influencing thymic tolerance and thymic involution, in turn, potentially affecting peripheral immune homeostasis (53, 54). In this context, our findings suggest that surgical removal of thymoma-bearing thymic tissue is accompanied by remodeling of peripheral immune communication. However, given the presence of thymoma and the absence of overt MG development in our patients, the present findings should not be interpreted as evidence that loss of thymic function directly induces autoimmune inflammation.

This study had several limitations. First, the number of patients was small, reflecting the rarity of anti-AChR antibody-positive individuals without clinical MG undergoing thymectomy. Accordingly, the present findings should be regarded as hypothesis-generating and require validation in larger independent cohorts. In addition, because differential expression was initially assessed using a mixed-effects model (NEBULA) with patient as a random effect, the random-effect variance could not be reliably estimated from only three donors, and the large number of cells does not compensate for this limited number of biological replicates. We therefore did not treat the cell-level statistics as confirmatory. To address this, we additionally performed donor-level paired pseudobulk analyses, in which the biological unit is the patient rather than the individual cell. The major changes, including the downregulation of antigen-presentation-related transcripts (e.g., CD74) and TNF in monocytes, were recapitulated at the pseudobulk level and were consistent in direction across patients, indicating that the principal findings are not driven by pseudoreplication (data not shown). In addition, although the major pathway-level findings were largely preserved after exclusion of immediate early/stress- and activation-associated genes, the Fc receptor-mediated stimulatory signaling pathway in CD56^dim^ NK cells was no longer significantly enriched. Therefore, this particular pathway-level finding should be interpreted cautiously, as it may have been influenced by the immediate early/stress-associated transcriptional signature. Second, several immunological changes observed in this study, including the reduction in naïve T-cell populations and the relative increase in NK cells after thymectomy, are consistent with previous reports and are therefore not themselves novel. The principal contribution of this study lies in the integrated single-cell characterization of these changes and the identification of coordinated alterations in transcriptional states and inferred intercellular communication. Third, the clinical backgrounds of the three patients were heterogeneous. Case 2 had breast cancer, although the clinical status of the breast cancer remained unchanged between the pre- and post-thymectomy sampling points. Case 3 had ulcerative colitis and received prednisolone at 5 mg/day at the postoperative sampling point. The activity of ulcerative colitis also remained clinically stable between the pre- and post-thymectomy sampling points. Although the prednisolone dose was within the range generally considered to approximate a physiological glucocorticoid-equivalent dose, an influence of these comorbidities and corticosteroid treatment on peripheral immune profiles cannot be completely excluded. Importantly, analyses excluding Case 3 showed that the overall direction of the major transcriptional and pathway-level changes was largely preserved in Cases 1 and 2, supporting the robustness of the principal observations (data not shown). Fourth, postoperative sampling intervals differed among the patients (22, 9, and 7 months in Cases 1, 2, and 3, respectively). Although all sampling points fell within the broad time window during which post-thymectomy MG has been reported to develop, the present study cannot distinguish stable thymectomy-associated immune remodeling from changes that may depend on the time elapsed after surgery. Longitudinal studies with standardized and multiple postoperative sampling points will be required to clarify the temporal dynamics of these immune alterations. Fifth, none of the patients developed overt MG during the observation period, consequently limiting direct causal inference regarding disease onset. Notably, although the anti-AChR antibody level increased postoperatively in Case 3, this patient did not develop overt MG during follow-up. Therefore, the relationship between the observed peripheral immune alterations, changes in anti-AChR antibody levels, and subsequent MG development remains unclear. Finally, functional validation experiments were not performed to directly assess the effects of post-thymectomy NK cells on T-cell or B-cell responses. Future studies incorporating larger cohorts, serial longitudinal sampling, patients who subsequently develop MG, and mechanistic *in vitro* or *in vivo* assays are therefore required to validate and clarify the functional significance of the immune alterations identified here.

In summary, thymectomy induces broad and coordinated remodeling of the peripheral immune system. This remodeling is characterized by a relative reduction in naïve T cell populations, activation-associated transcriptional changes in remaining T cells, a relative increase in the proportion of CD56^dim^ NK cells accompanied by transcriptional remodeling, transcriptional remodeling of monocytes, and rewiring of intercellular communication. Furthermore, it involves a shift in the dominant inflammatory signaling axis from TNF- and resistin-mediated pathways in the pre-thymectomy condition to IFNγ-dominated signaling after thymectomy. The coexistence of transcriptional changes in different directions highlights the dual nature of thymectomy-associated immune remodeling. Accordingly, the balance between these immune alterations, as well as the transition between these distinct signaling states, might partially contribute to the susceptibility to post-thymectomy MG and represent certain aspect of immune homeostasis following thymus removal.

## Data Availability

The datasets presented in this study can be found in online repositories. The names of the repository/repositories and accession number(s) can be found below: https://www.ncbi.nlm.nih.gov/, GSE330576.

## Supplementary material

Supplementary Figure 1 Identification of platelet-lineage cells using Azimuth annotation. Uniform manifold approximation and projection (UMAP) visualization of all PBMCs (n = 93,714) from three patients with thymoma before and after thymectomy. Cells annotated as "Platelet" by Azimuth are highlighted in red (n = 339, 0.36% of total); all other cells presented in **Figure 1B** are shown in grey.

Supplementary Figure 2 Differential gene expression analysis of additional T cell subsets following thymectomy. **(A)** Volcano plot showing differentially expressed genes (DEGs) in T helper type 2 (Th2) cells annotated by SingleR comparing post-thymectomy to pre-thymectomy samples. Differential expression analysis was performed using a negative binomial mixed model. Genes with FDR < 0.05 and |log2 fold change| > 0.25 were considered significant. The x-axis represents log2 fold change (post vs pre), and the y-axis represents −log10 (FDR). Red dots indicate genes upregulated after thymectomy, blue dots indicate genes downregulated after thymectomy, and gray dots indicate non-significant genes. Selected genes are labeled. **(B)** Volcano plot of DEGs in regulatory T cells (Tregs) annotated by SingleR, displayed using the same criteria as in **(A)**. **(C)** Volcano plot of DEGs in Mucosal-Associated Invariant T (MAIT) cells annotated by SingleR, displayed using the same criteria as in **(A)**.

Supplementary Figure 3 T cell receptor repertoire diversity before and after thymectomy. **(A)** Stacked bar plots showing T cell clonal size distributions for each sample before and after thymectomy. Clones are grouped according to clonal size: single (x = 1), small (1 < x ≤ 5), medium (5 < x ≤ 20), large (20 < x ≤ 100), and hyperexpanded (x > 100). Bars represent the proportion of total T cell receptor (TCR) clones within each category per sample. **(B)** Line plots showing Shannon diversity indices of the TCR repertoire in peripheral blood mononuclear cells before and after thymectomy for each patient. Each colored line represents an individual patient. The p value indicates the result of a paired comparison between pre- and post-thymectomy samples. **(C)** Shannon diversity indices of the TCR repertoire stratified by T cell subsets before and after thymectomy, including naïve CD4^+^ T cells, naïve CD8^+^ T cells, Mucosal-Associated Invariant T (MAIT) cells, follicular helper T cells, regulatory T cells, T helper type 1 (Th1) cells, T helper type 2 (Th2) cells, T helper type 17 (Th17) cells, Th1/Th17 cells, terminal effector CD8^+^ T cells, effector memory CD8^+^ T cells, and terminal effector CD4^+^T cells annotated by SingleR. Each line represents an individual patient, and p values indicate paired comparisons between pre- and post-thymectomy conditions for each subset.

Supplementary Figure 4 T cell receptor repertoire diversity assessed by inverse Simpson index before and after thymectomy. **(A)** Inverse Simpson diversity index of the T cell receptor (TCR) repertoire calculated from total PBMCs for each patient before (pre) and after (post) thymectomy. Each colored line represents an individual patient. Statistical comparison between pre- and post-thymectomy samples was performed using a paired t-test, and the corresponding p value is shown. **(B)** Inverse Simpson diversity index of the TCR repertoire stratified by T cell subsets, including naïve CD4^+^ T cells, naïve CD8^+^ T cells, Mucosal-Associated Invariant T (MAIT) cells, follicular helper T cells, regulatory T cells, T helper type 1 (Th1) cells, T helper type 2 (Th2) cells, T helper type 17 (Th17) cells, Th1/Th17 cells, terminal effector CD8^+^ T cells, effector memory CD8^+^ T cells, and terminal effector CD4^+^ T cells annotated by SingleR. Each panel shows pre- and post-thymectomy values for individual patients, indicated by colored lines. Paired t-test p values are displayed for each subset.

Supplementary Figure 5 Characterization of Natural Killer cell subsets. **(A)** Dot plot showing expression of representative marker genes across Natural Killer (NK) cell subsets annotated by Azimuth. NK cell subsets include NK_CD56dim, NK_CD56bright, and NK Proliferating cells. Dot size represents the percentage of cells expressing each gene, and dot color indicates the average scaled expression level. **(B)** Gene set enrichment analysis of Hallmark pathways in NK_CD56dim cells comparing post- versus pre-thymectomy samples. Pathways with false discovery rate (FDR) < 0.25 are shown. Dot size reflects gene set size, and dot color indicates −log10 (FDR). The normalized enrichment score (NES) is plotted on the x-axis.

Supplementary Figure 6 Differential Intercellular Communication Patterns Before and After Thymectomy. (**A)** Heatmap showing differential number of interactions between sender (rows) and receiver (columns) cell groups annotated by Azimuth in post vs pre conditions, computed by CellChat. Color indicates the relative change (post compared with pre). **(B)** Heatmap showing differential interaction strength (edge weight) between sender and receiver cell groups in post vs pre conditions. Color indicates the relative change in aggregated interaction strength. **(C)** Chord diagrams summarizing signaling pathways that were downregulated (left) or upregulated (right) after thymectomy. Each chord represents a ligand–receptor interaction between sender and receiver cell types, with chord width corresponding to the relative interaction strength. Colors indicate the originating (sender) cell types.

Supplementary Figure 7 Differential cell–cell communication networks of RESISTIN, TNF, and TGFβ signaling pathways before and after thymectomy. **(A)** Circle plots showing the RESISTIN signaling pathway network in pre-thymectomy (left) and post-thymectomy (right) samples. Nodes represent immune cell types annotated by Azimuth, and directed edges indicate ligand–receptor-mediated communication from sender to receiver cells. Edge thickness reflects the relative interaction strength (communication weight) computed by CellChat, and edge colors correspond to the sender cell type. **(B)** Circle plots depicting the TNF signaling pathway network in pre-thymectomy (left) and post-thymectomy (right) samples, visualized using the same conventions as in **(A)**. Changes in edge number and thickness indicate alterations in TNF-mediated intercellular communication following thymectomy. **(C)** Circle plots illustrating the TGFβ signaling pathway network in pre-thymectomy (left) and post-thymectomy (right) samples.
